# Linking miR-138 and USP10-P53 signaling

**DOI:** 10.18632/oncotarget.27260

**Published:** 2019-11-12

**Authors:** Ri Cui

**Keywords:** P53, USP10, miR-138, mutation

***News on:*** *A negative feedback regulatory loop between miR-138 and TP53 is mediated by USP10 by Luo et al. Oncotarget. 2019; 10:6288–6296. https://doi.org/10.18632/oncotarget.27275*

P53 is one of the best known tumor suppressors and is known as the “guardian of the genome”. Since discovery of P53, its function has been extensively studied and P53 network keeps growing in complexity. P53 is a transcription factor, regulates and associates with a number of genes to exert central role in genomic maintenance and tumor inhibition [1, 2]. In the normal condition, the expression level of P53 in the cells is relatively low, owing to E3 ubiquitin ligase Mdm2 mediated ubiquitin-proteasome degradation pathway. Mdm2 and other P53 ubiquitination associated proteins were phosphorylated by ATM (ataxia-telangiectasia mutated) result in degradation through inhibiting their interaction with USP7 (ubiquitin specific peptidase 7) / HAUSP (herpesvirus-associated ubiquitin-speciﬁc protease), thereby stabilizing P53 when cells under stress [3, 4]. In addition, USP10 directly deubiquitinates P53 subsequently increasing P53 expression levels [5]. *TP53* gene is most frequently mutated gene in human cancers, as in about 50% of human cancers have *TP53* deletion or mutation. Mutation of *TP53* gene and the resultant inactivation of P53 allow evasion of tumor cell death and rapid tumor progression. Accumulating evidence indicates that mutant P53 is abundantly expressed in the certain type of human cancers and contributes to tumor progression and resistance to anticancer therapy [6]. Importantly, USP10 could involve deubiquitination of both wild-type and mutant P53 thus was considered as either tumor suppressor or oncogene [5].

MicroRNAs (miRNAs) are a class of small endogenous non-coding RNAs with 19–25 nucleotides long. In general, microRNAs bind to the 3’-untranslated regions (3’-UTR) of mRNA in a sequence specific manner inducing mRNA degradation or repressing mRNA translation. miRNAs have tissue specific expression pattern, function as tumor suppressor or oncogene depend on tumor tissue type [7]. In 2002, Dr. Croce’s lab revealed for the first time that the potential importance of miRNAs in the diagnosis, prognosis and progression of a malignancy. They found that deletion of two miRNAs, miR-15a and miR-16-1 at chromosome 13q14 region, lead to development of indolent form of CLL (chronic lymphocytic leukemia) [8, 9].

In this context, Luo et al. found that USP10 is a direct target of miR-138. miR-138 overexpression inhibited both USP10 mRNA and protein expression levels. Since USP10 deubiquitinates and stabilizes P53, they further examined the effects of miR-138 on the expression level of P53 and observed that consistent down-regulation of P53 mRNA and protein levels after overexpressing miR-138. miR-138 overexpression also inhibited P53 dependent transcriptional activity by repressing USP10 and attenuated P53 dependent cell apoptosis and cell cycle arrest. By using Chip assay and Luciferase reporter assay, Luo et al. identified that P53 directly binds to the promoter region of miR-138 and negatively regulates miR-138 expression indicating that negative feedback regulatory loop between miR-138 and P53 [10].

P53 mutation status largely influences oncogenic or tumor suppressive functions of USP10 in cancer. Since miR-138 targets USP10, miR-138 could also work as either tumor suppressor or oncogene. In fact, miR-138 has been found to play a tumor suppressive role by targeting various oncogenes in non-small cell lung cancer, colorectal cancer, prostate cancer etc. In contrary, miR-138 plays oncogenic function by targeting tumor suppressor genes in glioblastoma [11]. These dual functions of miR-138 might be due to its different targets in different cancer tissue type. Another possibility is that miR-138 regulates either wild-type or mutated P53 by targeting USP10 as reported in this study. Further studies need to confirm that distinct role of miR-138 in tumorigenesis and P53 mutation status. Considering approximately half of human cancers with P53 mutation/deletion and regulatory axis of miR138 - USP10 - P53, miR-138 might be a promising therapeutic target upon identification of P53 mutation/deletion status.
